# Development and Validation of a Visual Grading Score of Disease Severity From Gait Videos in Genetic Peripheral Neuropathy

**DOI:** 10.1111/ene.70532

**Published:** 2026-03-20

**Authors:** Helena F. Pernice, Emanuele Piazza, Eleonora Asaad, Paul J. Wetzel, Katrin Hahn

**Affiliations:** ^1^ Klinik für Neurologie mit Experimenteller Neurologie Charité Universitätsmedizin Berlin Berlin Germany; ^2^ Berlin Institute of Health at Charité (BIH) Universitätsmedizin Berlin Berlin Germany; ^3^ Università degli studi di Verona Verona Italy

**Keywords:** Charcot–Marie–Tooth disease, gait, outcome assessment, health care, peripheral nervous system diseases, telemedicine

## Abstract

**Introduction:**

Monitoring individuals with peripheral neuropathies is challenging due to limited availability and time resources of neurologists, especially for rare genetic forms. This highlights the need for innovative methods to quantify disease severity efficiently and enable telemedical settings. In this study, we explored the feasibility of visual grading of gait disability as a novel approach for remote disease monitoring, using genetic neuropathies (Charcot Marie Tooth Disease, short CMT) as a model.

**Methods:**

We prospectively evaluated 53 participants with CMT using a battery of clinical examination scores, and patient reported outcome measures (PROMs), as well as semi‐standardized sagittal‐plane videos of participants walking a 4‐m runway for 5 ± 2 min cross‐sectionally, and developed the Visual Gait Assessment Scale in Polyneuropathy (vGASP) to grade the severity of gait abnormality.

**Results:**

Three independent raters evaluated the score as easily understandable and quick. The vGASP was internally consistent (inter‐class coefficient of 0.9) and valid when correlating with validated clinical outcome measures (Spearman's ρ = 0.6–0.8).

**Conclusions:**

The vGASP may offer a time‐effective solution for remote monitoring of patients in CMT and potentially other gait‐affecting polyneuropathies.

The Clinical Eye Score (CES) is a score to grade disease severity in peripheral neuropathy visually from videos of patients walking. It correlates strongly with state‐of‐the‐art outcome measures for peripheral neuropathy in patients with CMT. Therefore, it may provide a solution for digital or remote follow‐up examinations for adult patients with peripheral neuropathies.

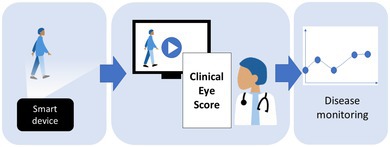

## 1 Introduction

Peripheral neuropathies affect 1% of the population and up to 20% of the elderly [1]. It usually manifests as distal to proximal progressive weakness and/or sensory deficit of the limbs, significantly impairing mobility and self‐sufficiency. Monitoring disease progression in peripheral neuropathies is essential for treatment decisions independent of etiology, ranging from autoimmune [2] to diabetic neuropathies [3]. However, measuring peripheral neuropathy is a challenge due to the usually slow progression, lack of objective biomarkers, and heterogeneity of the neuropathy itself [4]. While trained clinicians may detect disease severity and progression by the “naked eye”, quantification and comparison of the disease state is often difficult or cumbersome. Efforts to quantify disease severity are especially important in clinical or intervention trials: one of the types of neuropathies with the greatest need for such outcome measures is the most frequent genetically caused peripheral neuropathy, Charcot Marie Tooth disease (CMT), for which no causative therapy exists to date. CMT presents with a moderately homogenous phenotype and well depicts the progression of movement disability in peripheral neuropathy: it typically manifests in childhood or adolescence as impaired running and frequent ankle sprains, and continuously progresses to significant walking difficulties throughout life [5]. Foot drop is one of the most prominent features [6], causing a typical gait pattern described as “steppage” gait due to increased compensatory knee and hip lift [7]. With time, the disease progresses, and more proximal muscles get involved, causing a “waddling” gait [8]. Like for most other peripheral neuropathies, the key treatment for individuals with CMT is based on regular follow‐up to allow individualized (symptomatic) therapy, prevention and management of complications, as well as provision of aids to prolong mobility and self‐sufficiency [9]. Extensive efforts have been put into the development of clinical and patient reported outcome measures (COMs and PROMs, respectively) to perform such follow‐up assessments and allow educated clinical treatment decisions in peripheral neuropathy, such as CMT [10, 11, 12, 13, 14, 15, 16, 17]. However, performing such follow‐up examinations requires skilled neurologists, who are scarce and often only available after long waits and travels for patients, causing substantial costs in current healthcare settings [18]. Especially for patients living in rural or less developed regions, specialized care is often out of reach [19]. Recent care deficits in context of the COVID19‐pandemic have further shown the need for digital options in medicine [20]. In other neurological diseases, such as normal pressure hydrocephalus, Parkinson's disease, and multiple sclerosis, remote monitoring options via video‐assessment have been shown to be promising [21, 22, 23]. Likewise, a previous study using a virtual version of the CMT Examination score (CMTES) [17] has proven feasibility of remote care in CMT, but requires specific training of patient and healthcare professionalism [24]. Hence, despite the current efforts to increase telemedical care throughout Europe [25], generalizable tools to follow up peripheral neuropathy remotely are still missing. In this study, we aimed to fill this gap by developing an easy‐to‐use scale for remote evaluation of disease severity in peripheral neuropathy independent of patient or healthcare professional training. We used CMT as a model disease for peripheral neuropathy to validate this tool, given the representative clinical picture of a progressive peripheral neuropathy, as well as the paramount need for new care strategies, especially in this rare disease.

## 2 Methods

### 2.1 Patient cohort

Fifty‐three participants over 14 years old and with clinical or genetic diagnosis of CMT were prospectively recruited between 2022 and 2024 in the context of the CMT‐Registry including gait videos (CMT‐Registry study EA2/169/22). Approval for this study was given by the local ethics committee, and written informed consent was obtained before data collection by participants, as well as legal guardians of minor participants. All patients who presented to the CMT specialist clinic and agreed to the CMT‐registry study, including video recordings were included. The diagnosis of CMT was established via reported pathogenic genetic variant for any CMT subtype, or typical medical history (childhood onset slowly progressive peripheral neuropathy and/or positive family history for clinically highly similar presentation) without any other reason such as metabolic, toxic, traumatic, para/neoplastic, inflammatory, or other secondary neuropathy, which were ruled out by extensive laboratory testing and history taking. Genetic testing was performed using Sanger sequencing when the variant was known, or whole exome sequencing based panel analysis by either the Medizinisch Genetisches Zentrum (MGZ), Schlosserstraße 6, 80,335 München, or the local genetic department of Charité Universitätsmedizin Berlin. Potential selection bias was addressed by recruiting all eligible participants who agreed to video capture in the context of the CMT Registry study for all analyses. All examinations and data collection were performed by the same physician.

### 2.2 Clinical evaluation and nerve conduction studies

Neurological examination and nerve conduction studies were performed as described before [26], and included the Overall Neuropathy Limitation Scale (ONLS) [15], the neuropathy impairment Score (NIS) and NIS‐LL for only lower‐limb evaluation [10, 13], and the 10‐m walk test (10MWT) [27]. Additionally, we used the CMTES and the CMTNS in their original version, as version 2 was not available due to a lack of data on the radial nerve [28].

### 2.3 Patient reported outcome measures

Patient reported outcome measures (PROMs) were acquired using digital questionnaires or live acquisition during the clinic visits and included the Walk12 Score [29], the Overall Neuropathy Limitation Scale (ONLS) [15], and the Rasch‐modified Overall Neuropathy Disability Scale (R‐ODS) [14].

### 2.4 Video recording

**FIGURE 1:**
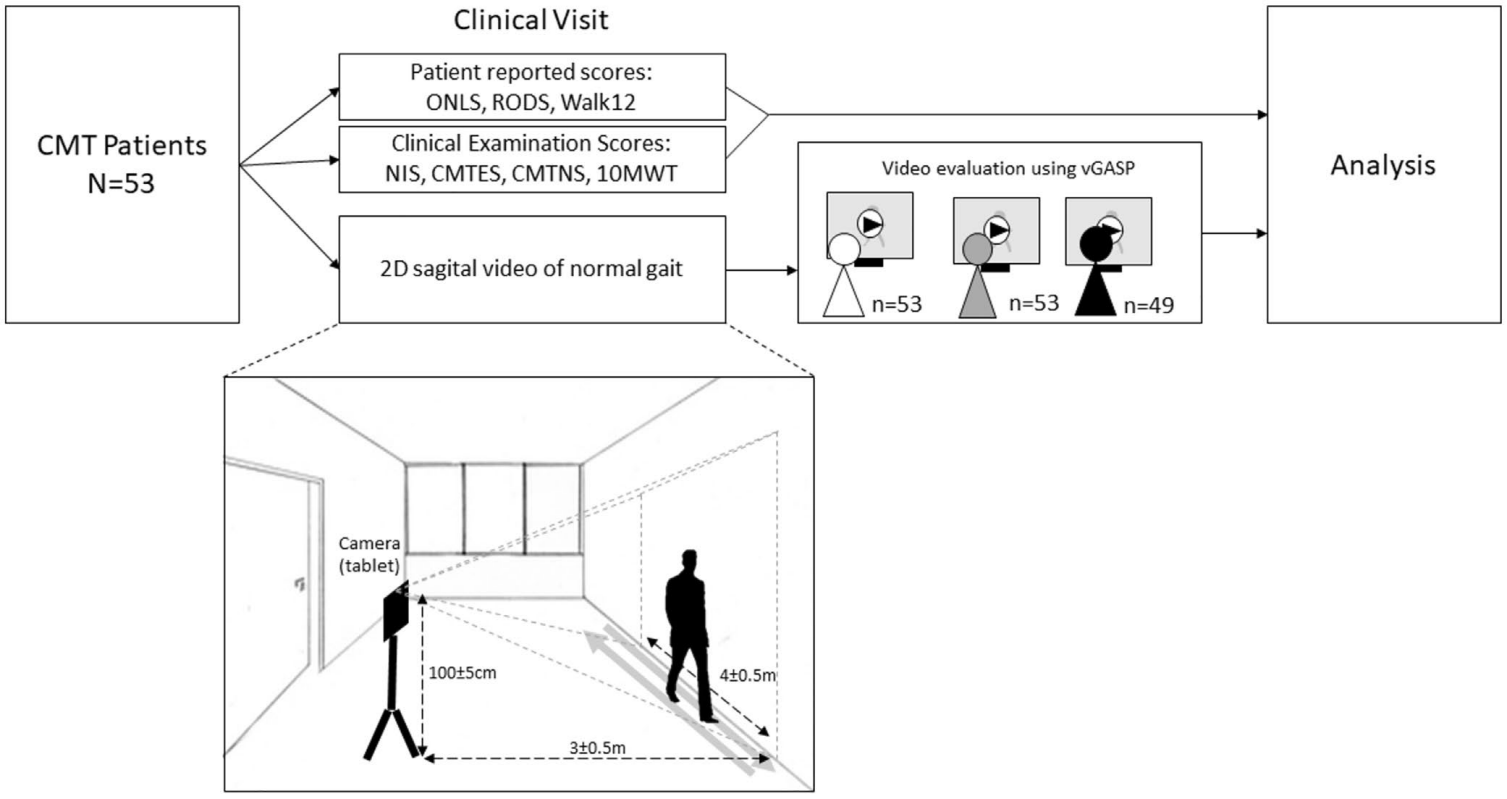
Workflow and setup for video‐recording. Patients were asked to walk from side to side on a 4 ± 0.5‐m runway in a room located at the outpatient clinic with a plain background. Camera distance and height are indicated (3 ± 0.5 m and 100 ± 5 cm, respectively). The camera was statically fixed on a tripod with a sagittal view of the patient.

All 53 subjects had sagittal plane videos of normal walk of a 4‐m runway (back and forth) for 5 ± 2 min, recorded as depicted in Figure 1, on the same day as clinical outcome measures and questionnaires (COMs and PROMs) were completed. Videos were taken using a Samsung Galaxy Table A8 (SM‐X200, 26,7 cm, 1920 x 1200 pixels, Android 11). Three participants recorded videos at home after receiving detailed instructions, all other 50 participants were recorded within the outpatient clinic setting. Here, medical personnel were close or present during the videotaping for safety. Participants were asked to walk without shoes and tight or no leg‐wear when possible. Participants who were unable to walk without shoes (i.e., orthopedic shoes) or needed walking support (i.e., crutches) used these for walking.

### 2.5 Development of the Visual Gait Assessment Scale in Polyneuropathy (vGASP)

The “Visual Gait Assessment Scale in Polyneuropathy” (vGASP) was developed as a structured clinical observation tool to aid the assessment of disease severity in polyneuropathy (on the model disease CMT). The scale consists of 12 items separated into 5 sections evaluating gait features, each of which is rated with scores between 0 (for no problem), 1 (for mild), 2 (for moderate), and 3 (for severe), or 1 (for mild/not compensatory), and 2 (for severe/compensatory). Points are then added up to a sum score between a minimum of 0 points, referring to no impairment, and a maximum of 26 points, representing very severe impairment. The single items for gait evaluation were developed using validated CMT COMs, other video assessment scores in neurological disease, and personal experience as references. A detailed description explaining the development of each item is depicted in Suppl. Table 1. The 5 sections evaluate (1) general gait (use of aids, speed, endurance, regularity), (2) leg movement (initial foot contact with the floor, knee/hip lift during swing phase, vaulting), (3) turn, (4) symmetry, and (5) upper body (arm swing, body posture). For symmetry evaluation, a negative score (−1) was allowed to compensate for points given for unilateral affection when there was only one affected and one healthy leg. More weight (up to 3 points) was given to the items “Aids”, “Initial foot contact”, and “knee/hip lift during swing phase”, as these items were considered to represent mobility impairment or the typical “steppage” gait most prominently [30].

## 3 Study Design

After collection of clinical data, PROMs, and videos, three independent neurologists were asked to rate patient videos using the Visual Gait Assessment Scale in Polyneuropathy (vGASP). The three raters represented different levels of neurological expertise: rater 1 was a first‐year neurology resident with no prior medical experience, rater 2 was a last‐year neurology resident just before the completion of neurological training and moderate prior experience with neuromuscular disease including peripheral neuropathies such as CMT, and rater 3 was an experienced senior neurologist with over 20 years of expertise in care of neuromuscular disease/peripheral neuropathy. All raters were blinded to any further patient data and had not seen any of the CMT patients previously. Raters were instructed in the scoring and its application as depicted in suppl. Table 1. Due to logistic and time restrictions, one rater only evaluated 49 videos. After internal validity analysis, we used the means of sum scores (rounded to full numbers) given by the three raters for each patient for further validity evaluation (referred to as “mean rounded sum score” in the result section). We used the STARD checklist when writing our report [31].

### 3.1 Statistical Analysis

Statistical analysis was performed with GraphPad Prism (Version 10, GraphPad, San Diego, CA, USA), and SPSS Statistics 30^th^ version. Reliability testing was done using Cronbach's alpha and interclass correlation (ICC); bias was tested using Bland–Altman plots and Wilcoxon signed rank test. Normal distribution was assessed using the evaluation of data histograms. Validity testing was performed using Spearman correlation testing as well as Kruskal‐Wallis test and Mann–Whitney‐U tests for comparison of groups. No adjusting for multiple testing was done when post hoc testing for pairs among groups of three was conducted when Kruskal‐Wallis testing had shown significant differences.

## 4 Results

### 4.1 CMT patient cohort

Demographics of participants are depicted in Table 1. We included a total of 53 individuals with a clinical or genetic diagnosis of CMT. The average age at the time of the visit was 41.9 years (range 16–80), 25 (47.2%) were female, and 15 (28.3%) used any kind of aids (including crutches, walkers, wheelchairs) continuously or sporadically. The mean age of disease onset was 17 years (range 1–49 years). While all participants fulfilled clinical diagnostic criteria for CMT as stated above, 22.6% of participants' genetic cause remained unsolved despite genetic testing using whole‐exome sequencing (WES). To elaborate on the impact of comorbidities in the cohort, structured medical history was taken using self‐reported questionnaires, revealing a prevalence of 23 (43.4%) of individuals with comorbidities. These included orthopedic problems (24.5%), thyroid disease (9.4%), diabetes mellitus (7.5%), and other diseases that may impact mobility, such as peripheral vascular disease and history of stroke (Table 1). To evaluate whether these diseases had a significant impact on the clinical outcome measure CMTES, CMTNSv1, and NIS, we compared sum scores of each COM in participants with and without each comorbidity. Here, no significant difference in any of the groups was detected (Suppl. Table 2). No adverse events occurred during video recording for patient evaluation.

**TABLE 1:** Demographics and basic clinical information of patient cohort. *Disease severity determined by age‐adapted CMTES.

Epidemiology of CMT cohort		
Number of patients	53	
Sex female (n, %)	25	47.2%
Use of Aids (n, %)	15	28.3%
Age in years (mean, range)	41.9	16–80
Age of reported disease onset in years (mean, range)	17	1–49
BMI (mean, stdv)	24.7	5.2
Use of Aids (n, %)	15	28.3%
Number of patients with mild/moderate/severe disease* (%)	9/34 / 10	17% / 64% / 19%
**Affected gene**	**n**	**%**
PMP22 (duplication)	11	20.8
PMP22 (deletion)	4	7.5
MPZ	4	7.5
GJB1	7	13.2
HSPB1	2	3.8
SORD	5	9.4
Unsolved	12	22.6
Other	7	13.2
*Other (all n = 1): LRSAM1, HARS, MORC2, HINT1, SH3TC2, NDRG, SPTLC2*		
**Comorbidities**	**n**	**%**
Patients with comorbidities	23	43.4
Diabetes mellitus	4	7.5
Peripheral vessel disease	1	1.9
Malignant disease	1	1.9
Autoimmune disease	3	5.7
Kidney disease	3	5.7
History of stroke	2	3.8
Cardiovascular disease	3	5.7
Orthopedic disorder	13	24.5
HIV	1	1.9
Thyroid disease	5	9.4
Depression	2	3.8
Other	9	17.0

*Other: Migraine, asthma, restless leg syndrome, hearing disorder, gastric ulcer, history of pneumothorax/*.

### 4.2 Usability and internal consistency of the Visual Gait Assessment Scale in Polyneuropathy

**FIGURE 2:**
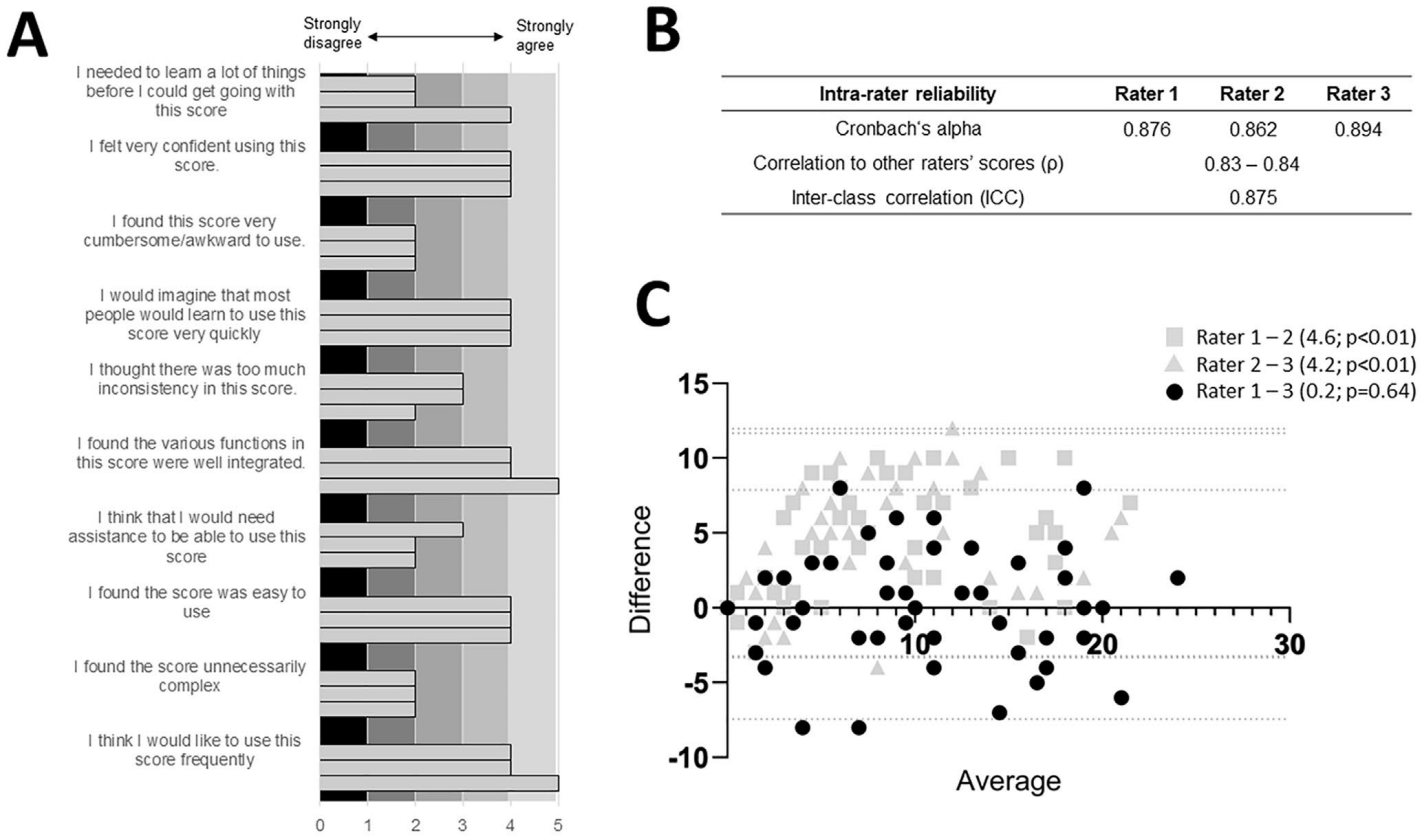
Usability and consistency of the Visual Gait Assessment Scale in Polyneuropathy. (A) Bar plots of all three raters' evaluation of the Visual Gait Assessment Scale in Polyneuropathy using the System Usability Scale. (B) Internal reliability testing. (C) Bland–Altman plot of raters to average. Legend depicts bias (first value) and *p*‐values according to Wilcoxon signed rank testing.

Three independent neurologists were asked to use the score to evaluate the included patient videos without any additional information on the participants. Three videos were shorter than one minute, and/or feet were partially cropped. In these videos, the items “endurance” and evaluation of foot posture were estimated best possible by raters from available information in videos. All raters then answered the System Usability Scale (SUS) (Figure 2, A) and generally evaluated the score as easy to understand, easy to use, consistent, and well‐representative. They needed a median time of 2 min to complete the score per subject. Internal (intra‐rater) consistency was good for all raters (Cronbach's alpha = 0.86–0.89), as well as between raters (inter‐rater, Cronbach's alpha = 0.93). We also found good correlation (Spearman's *r* = 0.80–0.84) and inter‐class reliability (ICC, 0.875) between the raters (Figure 2, B). Bland–Altman plots of sum scores between rater 2 to rater 1 and 3 revealed a systematic bias of rater 2 of 4.2 and 4.6 points, respectively, which was significant in Wilcoxon signed‐rank test (bias of rater 2 to rater 1 = 4.6, *p* < 0.01; bias of rater 2 to rater 3 = 4.2; p < 0.01; bias of rater 1 to rater 3 = 0.2, *p* = 0.64; Figure 2, C). Despite this bias of one rater over the others, we considered the inter‐rater reliability sufficient to use the average values of each item as a surrogate for further validity testing.

### 4.3 Correlation of the Visual Gait Assessment Scale in Polyneuropathy (vGASP) to Clinical Outcome Measures

For general validity, we correlated sum scores of the vGASP with sum scores of validated clinical COMs performed by a fourth, independent neurologist. We found age and presence of comorbidities correlated weakly but significantly with the mean rounded vGASP sum score, while there was no correlation with reported age of disease onset (Table 2, top). All clinical scores correlated with vGASP mean rounded sum scores, including the CMT Neuropathy Score Version 1 (CMTNSv1) and its sub‐score, the CMT Examination Score (CMTES), the Neuropathy Impairment Score (NIS) and its sub‐score for lower limbs (NIS‐LL), and the 10‐m walk test (10MWT; see Table 2, middle). Also, all PROMs correlated with the vGASP, especially the Walk12 (see Table 2, bottom).

**TABLE 2:** Validity testing by Spearman correlation of Visual Gait Assessment Scale in Polyneuropathy (vGASP) to demographic factors and clinical outcome measures.

vGASP mean rounded sum score correlation to	Spearman's ρ	95% confidence interval	P‐value	n
Age	034	0.07 to 0.57	0.01	53
Reported disease onset	0.00	−0.33 to 0.34	0.98	36
Comorbidities	0.32	−0.00 to 0.59	0.05	39
Clinical Outcome Measures				
CMTES	0.70	0.53 to 0.82	< 0.01	53
CMTNSv1	0.71	0.46 to 0.86	< 0.01	28
NIS	0.78	0.65 to 0.87	< 0.01	53
NIS‐LL	0.84	0.73 to 0.90	< 0.01	53
10MWT time (s)	0.76	0.36 to 0.92	< 0.01	14
10MWT steps (n)	0.56	0.00 to 0.86	0.05	13
Patient Reported Outcome Measures				
Walk12 test	0.76	0.58 to 0.87	< 0.01	40
ONLS	0.66	0.44 to 0.81	< 0.01	42
R‐ODS	−0.64	−0.81 to −0.39	< 0.01	35

To further explore which items of the vGASP were best suited to classify disease severity, we correlated different items to the CMTNSv1 or CMTES sum scores as previously described [32]. As depicted in Table 3, mean scores of all individual items of the vGASP correlated well with CMTES and CMTNSv1, except for the additional item for unilateral affection. However, this was expected since this item was only generated as an optional item to lessen the weight of points given for “asymmetry” if only one leg was affected (for more explanation, see Suppl. Table 1).

**TABLE 3:** Correlation of vGASP items with CMTNSv1 and CMTES.

	Correlation to CMTNSv1			Correlation to CMTES		
	Spearman ρ	p‐value	n	Spearman ρ	p‐value	n
Aids	0.64	< 0.01	53	0.50	< 0.01	28
Initial Foot Contact	0.55	< 0.01	53	0.59	< 0.01	28
Speed	0.68	< 0.01	53	0.70	< 0.01	28
Hip lift	0.60	< 0.01	53	0.68	< 0.01	28
Vaulting	0.67	< 0.01	53	0.71	< 0.01	28
Turn	0.64	< 0.01	49	0.66	< 0.01	27
Endurance	0.55	< 0.01	53	0.57	< 0.01	28
Regularity	0.70	< 0.01	53	0.77	< 0.01	28
Symmetry	0.68	< 0.01	53	0.78	< 0.01	28
Unilateral Affection	−0.25	0.08	50	−0.34	0.10	25
Arm swing	0.52	< 0.01	53	0.64	< 0.01	28
Posture	0.49	< 0.01	53	0.66	< 0.01	28

### 4.4 Discrimination of Disease Severity using the vGASP

**FIGURE 3:**
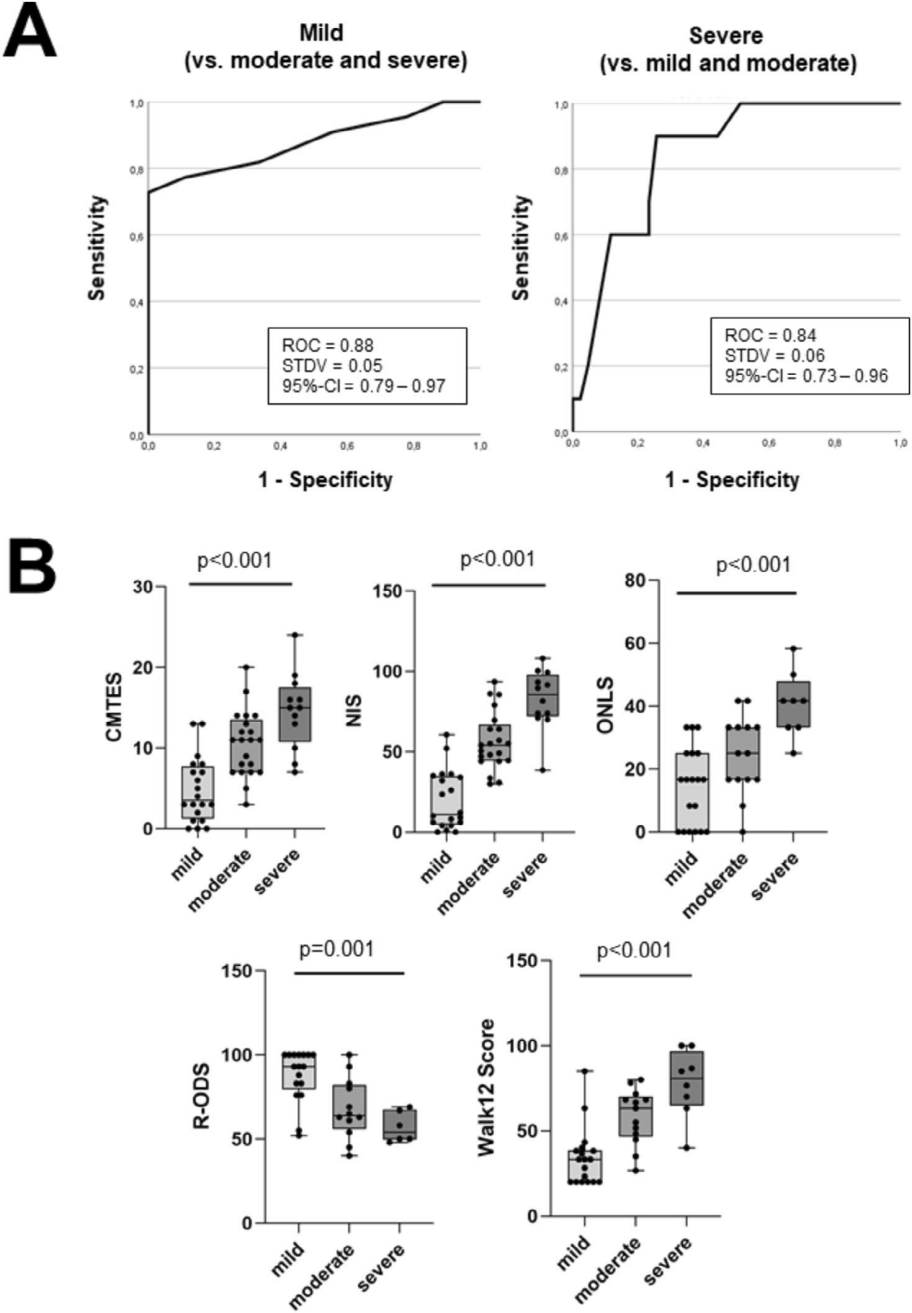
Discrimination of disease severity by the Visual Gait Assessment Scale in Polyneuropathy (vGASP). (A) Sensitivity and specificity ROC for age‐adapted CMTES classification. (B) Comparison of clinical outcome measures between groups classified by vGASP using cut‐offs defined by Youlden's index. CMTES = CMT Examination Score, NIS = Neuropathy Impairment Score, R‐ODS = Rasch modified Overall Disability Score, ONLS = Overall Neuropathy Limitation Scale. Statistics: (A) AUC = area under the curve, STDV = standard deviation, 95%‐CI = 95% confidence interval. (B) Kruskal‐Wallis test, *p*‐values shown on graphs.

We next asked the question whether the vGASP was sensitive and specific for discriminating between participants who were mildly, moderately, or severely affected according to the CMTES. For this test, we chose the CMTES for Receiver Operating Characteristic (ROC), since there was the highest number of available data for the CMTES (compared to other clinical outcome measures used in the study). Here, we found acceptable areas under the curve (AUCs) for differentiating mildly and severely affected individuals mild vs. moderate/severe: AUC = 0.88; standard deviation 0.05; 95% confidence interval 0.79–0.97; severe vs. mild/moderate: AUC 0.84; standard deviation 0.06; 95% confidence interval 0.73–0.96; Figure 3, A). We then used the Youlden's index to define cut‐offs for the vGASP (Cut‐off for mild: under 6 points, sensitivity = 1.00, specificity = 0.72; cut‐off for severe: over 15 points, sensitivity = 0.87, specificity = 0.87), and compared clinical scores between subject groups classified as mild, moderate, or severe using the CES. Here, sum scores of CMTES, NIS, ONLS, RODS, and Walk12 were all significantly worse in more affected individuals (CMTES: *n* = 53, median[mild] = 3.5, IQR = 6.5; median[moderate] = 11.0, IQR = 6.5; median [severe] = 15, IQR = 6.8, *p* < 0.01. NIS: *n* = 53; median[mild] = 11.0, IQR = 30.25; median[moderate] = 54.0, IQR = 22.5; median[severe] = 85.0, IQR = 26, *p* < 0.01. ONLS: *n* = 41, median[mild] = 16.7, IQR = 25; median[moderate] = 25.0, IQR = 16.7; median[severe] = 41.7, IQR = 14.6, *p* < 0.01. RODS: *n* = 35, median[mild =93.0, IQR = 29.5; median[moderate] = 25.0, IQR = 26.5; median[severe] = 47.9, IQR = 18.0, *p* < 0.0012. Walk12: *n* = 39, median[mild] = 33.3, ICR = 18.8; median[moderate] = 63.3, IQR = 23.3; median[severe] = 80.8, IQR = 31.7, *p* < 0.0001; see Figure 3, B).

## 5 Discussion

In this study, we developed and validated a new tool called the “Visual Gait Assessment Scale in Polyneuropathy” (vGASP) for remote estimation of disease severity from 2D videos of natural gait in individuals with peripheral neuropathy, recorded by standard commercial smart devices (tablets or smartphones), using an example cohort of participants diagnosed with CMT. The 12‐item score was evaluated as easy to use and well accepted by three independent neurologists and meant minimal effort for subjects. Our reliability analysis showed that items were internally consistent, showing highly similar evaluations among the three evaluators despite different levels of clinical experience, including neurologists at the beginning of their training, and without any further explanation. This suggests that the vGASP may be applicable to a wide range of neurologists and does not require special training or experience with rare diseases such as CMT.

Compared to other virtual measures for polyneuropathy, especially the virtual CMTES in the case of CMT, in which specifically trained personnel are needed [24], the vGASP was performed with no additional training of either raters or subjects. While other remote tools for evaluating neuropathy, such as smart sensors used in diabetic neuropathy also may not need active schooling of participants [33], the vGASP required no equipment beyond a smartphone camera, instead of complex technology or devices. We propose that this allows scalability to real‐world conditions in which affected individuals record videos at home. We recognize that the majority of videos in this study were recorded at the outpatient clinic with medical personnel close or present during recordings, which may not depict real‐life circumstances at the individuals' home [34]. Further studies testing the vGASP at the subjects' home will be necessary to evaluate the usability in a real‐world setting, with a more elaborate evaluation of telemedical concepts as well as data safety and privacy concerns.

In our validation analysis, we were able to show that vGASP scores correlated well with COMs and PROMs, and all (relevant) score items correlated well with the most widely used COM for CMT, the CMTES/CMTNS. Since CMT naturally progresses with age [35], the mild but significant correlation with age confirms an adequate evaluation of disease severity using the vGASP, and also the presence of comorbidities correlates with disease severity, which has been shown before in elderly populations [36]. While it must be taken into account that COMs used to control the validity of the vGASP do not perfectly represent disease severity independently [37], the combination of a wide range of COMs and PROMs used in this study in an attempt to determine the “ground truth” of disease severity approximates the “true” severity, and especially respects the individuals' disease experience and daily life function [38]. Not surprisingly, given that the vGASP focuses on walking abilities, vGASP sum scores correlated best with COMs focusing on lower legs, like the NIS‐LL [10] and the Walk12 [29].

Interestingly, both these measures, as well as the R‐ODS [14], were originally developed for other neurological disorders (diabetic neuropathy, multiple sclerosis, immune‐mediated neuropathies, respectively), and the ONLS [15] was validated in different types of neuropathy (including inflammatory, idiopathic, paraprotein‐associated, and genetic neuropathies). Since these scores all correlated well with the vGASP in our cohort, we propose that this new score will also be scalable from the CMT cohort tested here to other types of neuropathy.

An important limitation of the vGASP lies in the potential bias due to subjectivity of ratings: While the assessment is guided regarding features of gait abnormality, categories were not defined, and raters could judge freely whether an item was “mild” or “severe”. Such subjective bias was reflected in this study, as one rater systemically gave fewer points than the other two. The difficulty of determining reasons for such bias and eliminating them limits the general usability of the vGASP, especially in the context of clinical studies. However, almost all available COMs fundamentally depend on subjective scoring by the examiner (or patient). The current applicability of the vGASP for disease monitoring is limited since this study was performed cross‐sectionally and does not evaluate change over time, which is the clinically relevant outcome of any disease severity scale [39]. Also, since the vGASP is fundamentally based on gait, only ambulatory individuals can be evaluated, which excludes a significant amount of non‐ambulatory people with polyneuropathy from this method of remote care.

Future studies will be necessary to evaluate the usability of the vGASP in larger cohorts, including more uncontrolled videos by individuals taken at home and/or external cohorts, other types of neuropathies, as well as longitudinal assessment of disease progression. Furthermore, the potential applicability of this tool for screening of neuropathy may be feasible, suggesting further evaluation of the discriminative power of the COMs within cohorts with different gait abnormalities and different underlying diseases.

Taken together, our results suggest that remote disease evaluation by the “naked eye” guided by a simple scale is easily feasible, reliable, and valid in CMT, and we believe that this scale may also be usable in other peripheral neuropathies. In the future, the vGASP may serve as a tool for remote evaluation of disease severity in polyneuropathies, monitoring of treatment response allowing educated clinical decision making, and potentially polyneuropathy screening, in telemedicine as well as live assessments, saving significant amounts of time and resources and contributing to barrier‐free, spatially inclusive, and comprehensive care.

## Author Contributions

H.F.P., E.P., and K.H. designed the study. H.F.P., E.P., P.J.W., E.A., and K.H. collected the data. H.F.P. and E.P. analyzed the data. H.F.P. and E.P. wrote the manuscript. All authors read and approved the final manuscript.

## Funding

This study was supported by the Deutsche Gesellschaft für Muskelkranke (DGM) GmbH (grant number: Pe5/2).

## Conflicts of Interest

Helena Pernice has received personal funding from Deutsche Gesellschaft für Muskelkranke (DGM) GmbH and Berlin Institute of Health (BIH), as well as project funding, travel support, and speaker fees by Alnylam Pharmaceuticals Inc., unrelated to this article. Eleonora Asaad received financial reimbursement for travel support to attend scientific meetings by Amicus. Emanuele Piazza and Paul J. Wetzel declare they have no conflicts of interest. Katrin Hahn received financial reimbursement for consulting, advisory board activities, speaker fees, and/or contributions to congresses and travel support to attend scientific meetings by Akcea Therapeuticals Inc., Alnylam Pharmaceuticals Inc., Amicus, AstraZeneca, GSK, Hormosan, Takeda Pharmaceutical Inc., Pfizer Pharmaceuticals Inc., and Swedish Orphan Biovitrum Inc. and ViiV Healthcare GmbH, and research funding by the foundation Charité (BIH clinical fellow), Alnylam Pharmaceuticals Inc., and Pfizer Pharmaceuticals unrelated to this project.

## Supporting information


**Appendix S1:** Supporting Information.

## Data Availability

The data that support the findings of this study are available on request from the corresponding author. The data are not publicly available due to privacy or ethical restrictions.

## References

[1] W. Bronge , B. Lindholm , S. Elmståhl , and A. Siennicki‐Lantz , “Epidemiology and Functional Impact of Early Peripheral Neuropathy Signs in Older Adults from a General Population,” Gerontology 70, no. 3 (2024): 257–268, 10.1159/000535620. 38043521 PMC10911163

[2] A. L. Fisse , J. Motte , T. Grüter , M. Sgodzai , K. Pitarokoili , and R. Gold , “Comprehensive Approaches for Diagnosis, Monitoring and Treatment of Chronic Inflammatory Demyelinating Polyneuropathy,” Neurological Research and Practice 2, no. 1 (2020): 1–14, 10.1186/s42466-020-00088-8. 33324942 PMC7722337

[3] A. J. M. Boulton , “Diabetic Neuropathy: Classification, Measurement and Treatment,” Current Opinion in Endocrinology Diabetes 14, no. 2 (2007): 141–145, 10.1097/MED.0b013e328014979e. 17940432

[4] A. M. Rossor , M. E. Shy , and M. M. Reilly , “Are We Prepared for Clinical Trials in Charcot–Marie–Tooth Disease?,” Brain Research 1729 (2020): 146625, 10.1016/j.brainres.2019.146625. 31899213 PMC8418667

[5] D. Pareyson and C. Marchesi , “Diagnosis, Natural History, and Management of Charcot–Marie–Tooth Disease,” Lancet Neurology 8, no. 7 (2009): 654–667, 10.1016/S1474-4422(09)70110-3. 19539237

[6] C. J. Newman , M. Walsh , R. O'Sullivan , et al., “The Characteristics of Gait in Charcot–Marie–Tooth Disease Types I and II,” Gait & Posture 26, no. 1 (2007): 120–127, 10.1016/j.gaitpost.2006.08.006. 17010610

[7] M. Ferrarin , T. Lencioni , M. Rabuffetti , I. Moroni , E. Pagliano , and D. Pareyson , “Changes of Gait Pattern in Children with Charcot–Marie–Tooth Disease Type 1A: A 18 Months Follow‐up Study,” Journal of Neuroengineering and Rehabilitation 10, no. 1 (2013): 1–11, 10.1186/1743-0003-10-65. 23819439 PMC3707823

[8] S. S. Lee , H. J. Lee , J. M. Park , et al., “Proximal Dominant Hereditary Motor and Sensory Neuropathy with Proximal Dominance Association with Mutation in the TRK‐fused Gene,” JAMA Neurology 70, no. 5 (2013): 607–615, 10.1001/jamaneurol.2013.1250. 23553329

[9] B. Corrado , G. Ciardi , and C. Bargigli , “Rehabilitation Management of the Charcot–Marie‐Tooth Syndrome: A Systematic Review of the Literature,” Medicine 95, no. 17 (2016): e3278, 10.1097/MD.0000000000003278. 27124017 PMC4998680

[10] V. Bril , “NIS‐LL: The Primary Measurement Scale for Clinical Trial Endpoints in Diabetic Peripheral Neuropathy,” European Neurology 41, no. SUPPL. 1 (1999): 8–13, 10.1159/000052074. 10023123

[11] N. E. Johnson , C. Heatwole , P. Creigh , et al., “The Charcot Marie Tooth Health Index Evaluation of a Patient‐Reported Outcome,” Annals of Neurology 84 (2018): 225–233. 30014533 10.1002/ana.25282 PMC6168418

[12] J. Burns , R. Finkel , T. Estilow , et al., “Development, Reliability, and Validity of the Charcot–Marie–Tooth Disease Pediatric Scale (CMTPedS),” Journal of Foot and Ankle Research 4, no. S1 (2011): 6–7, 10.1186/1757-1146-4-s1-o12.21294889

[13] P. J. B. Dyck , A. González‐Duarte , L. Obici , et al., “Development of Measures of Polyneuropathy Impairment in hATTR Amyloidosis: From NIS to mNIS + 7,” Journal of the Neurological Sciences 405, no. August (2019): 116424, 10.1016/j.jns.2019.116424. 31445300

[14] S. I. Van Nes , E. K. Vanhoutte , P. A. Van Doorn , et al., “Rasch‐built Overall Disability Scale (R‐ODS) for Immune‐mediated Peripheral Neuropathies,” Neurology 76, no. 4 (2011): 337–345, 10.1212/WNL.0b013e318208824b. 21263135

[15] R. C. Graham and R. A. C. Hughes , “A Modified Peripheral Neuropathy Scale: The Overall Neuropathy Limitations Scale,” Journal of Neurology, Neurosurgery, and Psychiatry 77, no. 8 (2006): 973–976, 10.1136/jnnp.2005.081547. 16574730 PMC2077620

[16] K. Eichinger , J. Burns , K. Cornett , et al., “The Charcot–Marie‐Tooth Functional Outcome Measure (CMT‐FOM),” Neurology 91 (2018): e1381–e1384, 10.1212/WNL.0000000000006323. 30232254 PMC6177280

[17] S. M. Murphy , D. N. Herrmann , M. P. McDermott , et al., “Reliability of the CMT Neuropathy Score (second version) in Charcot–Marie–Tooth Disease,” Journal of the Peripheral Nervous System 16, no. 3 (2011): 191–198, 10.1111/j.1529-8027.2011.00350.x. 22003934 PMC3754828

[18] E. Schorling , S. Thiele , L. Gumbert , et al., “Cost of Illness in Charcot–Marie‐Tooth Neuropathy: Results from Germany,” Neurology 92, no. 17 (2019): e1–e11, 10.1212/WNL.0000000000007376. 30918088

[19] F. H. Chen , A. L. Hartman , M. C. V. Letinturier , et al., “Telehealth for Rare Disease Care, Research, and Education Across the Globe: A Review of the Literature by the IRDiRC Telehealth Task Force,” European Journal of Medical Genetics 72 (2024): 104977, 10.1016/j.ejmg.2024.104977. 39374775

[20] V. Patterson , “Neurological Telemedicine in the COVID‐19 Era,” Nature Reviews. Neurology 17, no. 2 (2021): 73–74, 10.1038/s41582-020-00438-9. 33257883 PMC7703718

[21] F. Gholami , D. A. Trojan , J. Kovecses , W. M. Haddad , and B. Gholami , “A Microsoft Kinect‐Based Point‐of‐Care Gait Assessment Framework for Multiple Sclerosis Patients,” IEEE Journal of Biomedical and Health Informatics 21, no. 5 (2017): 1376–1385, 10.1109/JBHI.2016.2593692. 27455529

[22] M. Ishikawa , S. Yamada , and K. Yamamoto , “Agreement Study on Gait Assessment using a Video‐assisted Rating Method in Patients with Idiopathic Normal‐pressure Hydrocephalus,” PLoS One 14, no. 10 (2019): 1–12, 10.1371/journal.pone.0224202. PMC6812866 31648232

[23] K. Eguchi , I. Takigawa , S. Shirai , et al., “Gait Video‐based Prediction of Unified Parkinson's Disease Rating Scale Score: A Retrospective Study,” BMC Neurology 23, no. 1 (2023): 1–11, 10.1186/s12883-023-03385-2. 37798685 PMC10552271

[24] V. Prada , M. Laurà , R. Zuccarino , M. M. Reilly , and M. E. Shy , “Virtual Charcot–Marie‐Tooth Examination Score: A Validated Virtual Evaluation for People with Charcot–Marie–Tooth Disease,” Neurology Clinical Practice 12, no. 5 (2022): E98–E104, 10.1212/CPJ.0000000000200070. 36380896 PMC9647800

[25] B. León‐Salas , Y. González‐Hernández , D. Infante‐Ventura , et al., “Telemedicine for Neurological Diseases: A Systematic Review and Meta‐Analysis,” European Journal of Neurology 30, no. 1 (2023): 241–254, 10.1111/ene.15599. 36256522

[26] H. F. Pernice , A. L. Knorz , P. J. Wetzel , et al., “Neurological Affection and Serum Neurofilament Light Chain in Wild Type Transthyretin Amyloidosis,” Scientific Reports 14, no. 1 (2024): 1–10, 10.1038/s41598-024-60,025-6. 38698025 PMC11066119

[27] N. Hadouiri , I. Fournei , C. ThauVin‐Robinet , A. Jacquin‐Piques , P. Ornetti , and M. Gueugnon , “Walking test Outcomes in Adults with Genetic Neuromuscular Diseases: A Systematic Literature Review of Their Measurement Properties,” European Journal of Physical and Rehabilitation Medicine 60, no. 2 (2024): 257–269, 10.23736/s1973-9087.24.08095-X. 38300152 PMC11114158

[28] M. E. Shy , J. Blake , K. Krajewski , et al., “Reliability and Validity of the CMT Neuropathy Score as a Measure,” Published online 64, no. 7 (2005): 1209–1214, 10.1212/01.WNL.0000156517.00615.A3. 15824348

[29] R. C. Graham and R. A. C. Hughes , “Clinimetric Properties of a Walking Scale in Peripheral Neuropathy,” Journal of Neurology, Neurosurgery, and Psychiatry 77, no. 8 (2006): 977–979, 10.1136/jnnp.2005.081497. 16574732 PMC2077614

[30] E. Wojciechowski , A. Sman , K. Cornett , et al., “Gait Patterns of Children and Adolescents with Charcot–Marie–Tooth Disease,” Gait & Posture 56 (2017): 89–94, 10.1016/j.gaitpost.2017.05.005. 28527386

[31] P. M. Bossuyt , J. B. Reitsma , D. E. Bruns , et al., “STARD 2015: An Updated List of Essential Items for Reporting Diagnostic Accuracy Studies,” BMJ 351, no. October (2015): 1–9, 10.1136/bmj.h5527. PMC4623764 26511519

[32] G. Coghe , M. Pau , E. Mamusa , et al., “Quantifying Gait Impairment in Individuals Affected by Charcot–Marie–Tooth Disease: The Usefulness of Gait Profile Score and Gait Variable Score,” Disability and Rehabilitation 42, no. 5 (2020): 737–742, 10.1080/09638288.2018.1506946. 30334469

[33] B. Najafi and R. Mishra , “Harnessing Digital Health Technologies to Remotely Manage Diabetic Foot Syndrome: A Narrative Review,” Medicina (Lithuania) 57, no. 4 (2021): 377, 10.3390/medicina57040377. PMC8069817 33919683

[34] L. P. Serrano , K. C. Maita , F. R. Avila , et al., “Benefits and Challenges of Remote Patient Monitoring as Perceived by Health Care Practitioners: A Systematic Review,” Permanente Journal 27, no. 4 (2023): 100–111, 10.7812/TPP/23.022. 37735970 PMC10730976

[35] M. E. Shy , L. Chen , M. E. R. Swan , et al., “Neuropathy Progression in Charcot–Marie–Tooth Disease Type 1A,” (2008), https://www.neurology.org. 10.1212/01.wnl.0000297553.36441.ce 18227419

[36] M. Cesari , G. Onder , A. Russo , et al., “Comorbidity and Physical Function: Results from the Aging and Longevity Study in the Sirente Geographic Area (iISIRENTE Study),” Gerontology 52, no. 1 (2006): 24–32, 10.1159/000089822. 16439821

[37] G. Piscosquito , M. M. Reilly , A. Schenone , et al., “Responsiveness of Clinical Outcome Measures in Charcot–Marie–Tooth Disease,” European Journal of Neurology 22, no. 12 (2015): 1556–1563, 10.1111/ene.12783. 26227902

[38] R. Mercieca‐Bebber , M. T. King , M. J. Calvert , M. R. Stockler , and M. Friedlander , “The Importance of Patient‐reported Outcomes in Clinical Trials and Strategies for Future Optimization,” Patient Related Outcome Measures 9 (2018): 353–367, 10.2147/prom.s156279. 30464666 PMC6219423

[39] B. Middel and E. Van Sonderen , “Statistical Significant Change Versus Relevant or Important Change in (quasi) Experimental Design: Some Conceptual and Methodological Problems in Estimating Magnitude of Intervention‐related Change in Health Services Research,” International Journal of Integrated Care 2, no. 4 (2002): e15, 10.5334/ijic.65. 16896390 PMC1480399

