# Supplementary material for: Development and Validation of a Visual Grading Score of Disease Severity From Gait Videos in Genetic Peripheral Neuropathy
Source: Eur J Neurol. 2026 Mar 20;33(3):e70532. doi: 10.1111/ene.70532 (PMC13093799; doi:10.1111/ene.70532)
Supplement: Supplementary file 1 — Appendix S1: Supporting Information. [file ENE-33-e70532-s001.docx]

**Supplemental data**

|  | **Item** | **Evaluation** |
| --- | --- | --- |
|  | **General gait** |  |
| **1** | **Aid** | This item has been developed in accordance with the ONLS [9], considering the use of both crutches/sticks and orthoses during walking; however, the unilateral or bilateral use of such devices is not critical for this specific item (see item; 'Symmetry' and 'Unilateral'). |
| **2** | **Speed** | Speed is a fundamental aspect of gait function in healthy and diseased [35,36]. Furthermore, in peripheral neuropathy, speed is relevantly affected by sensory loss, an important aspect of most CMT patients [37]. |
| **3** | **How does gait speed chance after 1 min?** | We developed this item focusing on the role of sustained physical activity in chronic neurologic diseases affecting gait based on previous findings in stroke [35]. |
| **4** | **How regular/flowing is the patient’s gait? Are steps the same size and speed?** | This item was developed to consider regularity of gait pattern, a significant factor in large- and small-fiber neuropathy [38,39]. |
|  | **Leg movement** |  |
| **5** | **Initial foot contact** | This item is adapted from the Visual Gait Assessment Scale in hemiplegic cerebral palsy [40]: foot drop is a typical sign of distal weakness in CMT, with consequent impaired foot contact at the beginning of stance phase. |
| **6** | **Knee/hip lift during swing phase** | Foot drop causes knee lift as a compensatory mechanism to avoid tripping over toes [6]. In later stages, a more proximal involvement may lead to hip lift impairment, i.e., waddling gait [41]. |
| **7** | **Vaulting** | The item focuses on the pattern described by Ferrarin et al. in children with CMT [6]. |
|  | **Turn** |  |
| **8** | **How fluid/sure does the patient's movement look when turning?** | As described for other neurological disease, turning is an important aspect of gait steadiness and consequent fall risk [42]. |
|  | **Symmetry** |  |
| **9** | **How symmetric do patient steps look while walking, comparing right and left lower limbs movements?** | This item was developed in respect to the importance of asymmetric lower limb involvement in neuromuscular disorder [43]. |
| **9 a)** | **Is one leg more affected than the other?** | This item is added to compensate a possible bias: considering item 9 alone, cases with severe symmetric involvement would have a lower score than cases with only one-sided involvement (bilateral symmetric steppage gait vs unilateral foot drop) |
|  | **Upper body** |  |
| **10** | **Do patients’ arm swing naturally with the contralateral leg during gait?** | Arm swing is an important aspect of human gait [44], and a known aspect in neurological diseases such as Parkinson’s disease with a relevant impact on gait quality [45]. |
| **11** | **Upper body posture/head** | The impact of body posture has been described in different gait disorder and neurological disease [46,47]. As also in CMT, balance problems may be compensated by body posture and gaze, we included this item. |
|  | **Sum** |  |

**Suppl. Table 1**. Development of the single items of the Visual Gait Assessment Scale in Polyneuropathy.

| **Score** | **Comorbidities (median, range)** | **No comorbidities (median, range)** | **p-value** |
| --- | --- | --- | --- |
| CMTES | 11 (0-24) | 8 (0-18) | 0,55 |
| CMTNSv1 | 7 (2-20) | 10 (0-23) | 0,60 |
| NIS | 44 (0-108) | 48 (0-100) | 0,88 |
| CES | 9 (0-22) | 7 (1-18) | 0,72 |

**Suppl. Table 2**: Comparison of disease scores between patients with and without comorbidities. CMTES = CMT Examination Score; CMTNSv1 = CMT Neuropathy Score Version 1; NIS = Neuropathy Impairment Score; vGASP = Visual Gait Assessment Scale in Polyneuropathy. Statistics: Mann-Whitney-and CMTES.
